# Biochemical, Histopathological and Morphological Profiling of a Rat Model of Early Immune Stimulation: Relation to Psychopathology

**DOI:** 10.1371/journal.pone.0115439

**Published:** 2015-01-20

**Authors:** Anna Kubesova, Hana Tejkalova, Kamila Syslova, Petr Kacer, Jana Vondrousova, Filip Tyls, Michaela Fujakova, Tomas Palenicek, Jiri Horacek

**Affiliations:** 1 Prague Psychiatric Center, Prague, Czech Republic; 2 National Institute of Mental Health, Klecany, Czech Republic; 3 Third Faculty of Medicine, Charles University, Prague, Czech Republic; 4 Institute of Chemical Technology, Prague, Czech Republic; University of Nebraska Medical Center, UNITED STATES

## Abstract

Perinatal immune challenge leads to neurodevelopmental dysfunction, permanent immune dysregulation and abnormal behaviour, which have been shown to have translational validity to findings in human neuropsychiatric disorders (e.g. schizophrenia, mood and anxiety disorders, autism, Parkinson’s disease and Alzheimer’s disease). The aim of this animal study was to elucidate the influence of early immune stimulation triggered by systemic postnatal lipopolysaccharide administration on biochemical, histopathological and morphological measures, which may be relevant to the neurobiology of human psychopathology. In the present study of adult male Wistar rats we examined the brain and plasma levels of monoamines (dopamine, serotonin), their metabolites, the levels of the main excitatory and inhibitory neurotransmitters glutamate and γ-aminobutyric acid and the levels of tryptophan and its metabolites from the kynurenine catabolic pathway. Further, we focused on histopathological and morphological markers related to pathogenesis of brain diseases - glial cell activation, neurodegeneration, hippocampal volume reduction and dopaminergic synthesis in the substantia nigra. Our results show that early immune stimulation in adult animals alters the levels of neurotransmitters and their metabolites, activates the kynurenine pathway of tryptophan metabolism and leads to astrogliosis, hippocampal volume reduction and a decrease of tyrosine hydroxylase immunoreactivity in the substantia nigra. These findings support the crucial pathophysiological role of early immune stimulation in the above mentioned neuropsychiatric disorders.

## Introduction

Negative environmental insults occurring during the intrauterine or early postnatal period may increase the risk of developing neuropsychiatric disorders in adulthood [1]. In recent years, attention has been focused on early immune stimulation caused by a maternal or perinatal infection. In various animal models early bacterial immune stimulation has been induced by administration of endotoxin, lipopolysaccharide (LPS) [2]. LPS is a part of the outer membrane of Gram-negative bacteria with a strong ability to induce an immune response [3]. LPS is recognized by Toll-like receptor 4 (TLR4) which is expressed in the central nervous system mainly on microglia and on other cell types in lower levels [4–6]. Activation of TLR4 leads to a cascade of steps that results in a release of pro-inflammatory and anti-inflammatory cytokines [3]. Immune challenge experienced in early postnatal days leads to a permanent immune dysregulation and alterations in the hypothalamic-pituitary-adrenal axis [7, 8].

Early immune stimulation might be one of underlying environmental factors which can predispose individuals to develop schizophrenia [9]. In our previous experiments, postnatal LPS treatment led to psychotic-like changes in behaviour and an increase of circulating cytokines in adult rats. These changes were normalized by the administration of clozapine [10–12]. Psychotic-like behaviour as well as anxiety-like, autistic-like and depression-like behaviour in adult animals perinatally treated with LPS has also been demonstrated in other studies [13–17].

Although the perinatal immune challenge (modelling both pre- and postnatal infection in humans) has been associated with the development of diverse neuropsychiatric disorders, little is known about its impact on biochemical actions in the adult brain. Histopathological changes, which might underlie the modelled behavioural phenotypes, have been investigated in several studies but the results are still contradictory [18–21].

Our study was designed to elucidate the influence of early immune stimulation on biochemical and histopathological changes in an adult rat brain and their potential relation to human psychopathology. Firstly, with respect to the role of major neurotransmitter systems in affective and psychotic disorders, we determined the levels of monoamines (dopamine, serotonin) and their metabolites, and the levels of the main excitatory and inhibitory neurotransmitters glutamate and γ-aminobutyric acid (GABA) in the brain. The activation of the kynurenine pathway of tryptophan metabolism has been associated with various neuropsychiatric disorders [22], therefore we mapped the brain levels of tryptophan and its metabolites from the kynurenine catabolic pathway regarding the effect of proinflammatory cytokines on the enzymes of this pathway. Since some of the neurotransmitters and metabolites do not cross the blood brain barrier (BBB) but in human subjects are determined from blood, we also measured their plasma levels. Secondly, we detected histopathological markers related to pathogenesis of brain diseases such as the ionized calcium-binding adaptor molecule (Iba-1, microglial marker upregulated during activation of these cells), glial fibrillary acidic protein (GFAP, astroglial marker), Fluoro-Jade B (marker of neurodegeneration) and Hoechst 33258 (marker of cell nuclei). Thirdly, to evaluate the effect of early immune stimulation on brain morphology, we measured the hippocampal volume, whose reduction is consistently described in patients with mental disorders [23–27]. The hypothesis of altered dopaminergic activity has been complemented by subsequent evaluation of tyrosine hydroxylase (TH) immunoractivity in the substantia nigra (SN) to elucidate the interaction between dopamine levels and the number of dopaminergic cells.

We expected an alteration in brain and plasma levels of neurotransmitters and metabolites and the activation of the kynurenine pathway of tryptophan metabolism. We assumed that early immune stimulation leads to a prolonged activation of glial cells, neurodegeneration, and hippocampal volume reduction.

## Methods

### Animals and housing

All of the experiments were carried out on male Wistar/Hann rats (Velaz Ltd., Czech Republic). Male rat pups remained in the breeding plastic cages with their mothers until PD 28 and were then divided into groups of three or four animals per cage. The animal facility was air conditioned at a standard room temperature and humidity with a normal 12h light/dark cycle. The rats had unlimited access to a standard diet and water. All procedures utilized in this article comply with the Czech Government Requirements under the Policy of Humans Care of Laboratory Animals and with regulations of the Ministry of Agriculture of the Czech Republic (No. 419/2012) and obtained statement of the Expert Committee for experimental animal protection (3^rd^ Faculty of Medicine, Charles University in Prague) and were approved by the Committee for Care and Use of Laboratory Animals of Ministry of Health (MHCR, No. 17/2012).

### Postnatal LPS treatment

From PD 5 to PD 9, the rat pups were each day briefly removed from their home cages, weighed, and administered intraperitoneally with either 2 mg/kg/day LPS (Escherichia coli, serotype 026:B6; Sigma–Aldrich) or an equivolume of non-pyrogenic 0.9% saline as described previously [10]. The animals were allocated to treatment groups randomly.

### Determination of neurotransmitters and metabolites in brain and plasma

**Figure 1:**
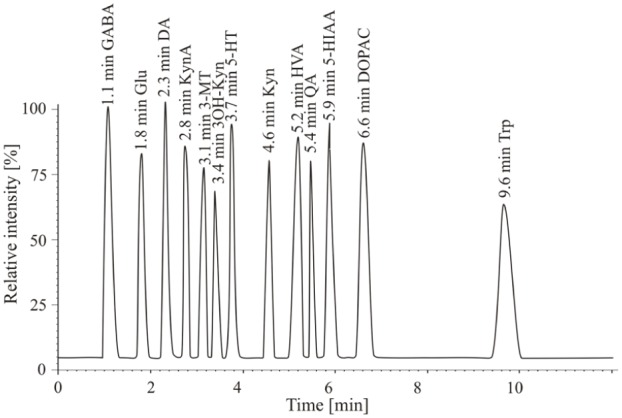
Chromatographic separation of the subject analytes.

The adult animals (n = 19–24/group) were euthanized by decapitation at PD 90–95. The collected brain tissue (striatum, hippocampus and prefrontal cortex) and plasma were immediately frozen at -80ºC until further processing. The analytical method used for qualitative and quantitative determination of neurotransmitters consisted of a pre-treatment step i.e. extraction by a mixed solution of acetonitrile, hydrochloric acid (c = 0.1 M) and EDTA (c = 27 M) at a ratio of 5:4:1 v / v / v, to separate and concentrate analytes (dopamine (DA), 3,4-dihydroxyphenylacetic acid (DOPAC), 3-methoxytyramine (3-MT), homovanilic acid (HVA), serotonin (5-HT), 5-hydroxyindolacetic acid (5-HIAA), glutamate (GLU), γ-aminobutyric acid (GABA), tryptophan (TRP), kynurenine (KYN), kynurenic acid (KYNA), 3-hydroxykynurenine (3-OH-KYN) and quinolinic acid (QUIN)) from the brain tissue or plasma, and a detection step using liquid chromatography combined with electrospray ionization tandem mass spectrometry (UHPLC–ESI-MS/MS). The analytic system consists of an Accela 1250 pump, Accela autosampler and a TSQ Vantage mass spectrometer (Thermo Scientific, USA). The analytes were separated on Kinetex C18 100 mm×2.1mm x 1.7um and mobile phase (solvent A: aqueous solution of acetic acid (pH 2); solvent B: methanol) in gradient elution at a flow rate of 300ul/min. The HPLC elution program was as follows: 5% B (2 min) → 30% B (linear increase in 1 min) → 30% B (5 min) → 5% B (linear decrease in 1 min) → 5% B (3 min). The column temperature was maintained at 25°C. The injection volume was 5 ul. The mass spectrometer equipped with an electrospray ion source was used for detection of DA, 5-HT, GABA, 3-MT, TRP, KYN, KYNA and 3-OH-KYN in the positive ionization mode (ESI^+^), and GLU, HVA, DOPAC, 5-HIAA and QUIN were analyzed in the negative ionization mode (ESI^-^). The selective reaction monitoring (SRM) mode was used. The details of the method were described elsewhere [28]. The chromatographic separation of the analytes is depicted in Fig. 1.

### Tissue preparation and histology

The adult animals (n = 8/group) were weighed and anesthetized with isofluran and perfused transcardially with Ringer solution followed by 4% paraformadehyde in Ringer solution at PD 93–97. The brains were removed and fixed overnight in 4% paraformaldehyde. The brain samples were cryoprotected in 30% sucrose in Ringer solution and sectioned at a 40μm thickness on a cryostat. The sections were stored at -40ºC in an antifreeze solution (sodium phosphate dibasic, glycerol, ethylene glycol) until further processing.

The tissue sections were stained with 0.1% Toluidine Blue (Nissl; Sigma Aldrich, USA) for routine histological examination and stereological evaluation of the hippocampal volume. The hippocampal volume was obtained using Cavalieri’s principle in Stereo Investigator software (MBF Bioscience, Inc., USA) and a light microscope (Olympus BX51). Nineteen to twenty-one sections (40μm) from the 1-in-6 series from the beginning of the hippocampus to the end of the CA3 region (from 1.7 to 6.1 mm dorsal from bregma according to a stereological atlas of the rat brain) [29] were analyzed.

The extent of neurodegeneration was determined using a neurodegeneration marker Fluoro-Jade B (Millipore/Merck KGaA, Germany) and a cell nuclei marker Hoechst 33258 (Sigma Aldrich, USA). Slides were evaluated under a fluorescence microscope (Zeiss Axio Imager Z1).

### Immunohistochemistry

Immunohistochemistry was performed on free-floating tissue sections according to standard procedures. The sections were briefly rinsed in Tris-Triton and incubated overnight at 4ºC with the primary antibody polyclonal rabbit anti-Iba1 (1:3000, Wako Pure Chemical Industries, Ltd., Japan, Cat.No. 019–19741), polyclonal rabbit anti-GFAP (1:5000, DAKO Denmark A/S, Denmark, Cat.No. Z0334) or polyclonal rabbit anti-TH (1:2000, Millipore/Merck KGaA, Germany, Cat.No. AB152). Primary antibodies were recognized by the biotinylated goat anti-rabbit IgG secondary antibody (1:300, Jackson ImmunoResearch Laboratories Inc., USA, Cat.No. 111–065–003), which was detected by Avidin-Peroxidase-Complex (ABC) solution (Vectastain Elite kit standart, Vector Laboratories, Inc., USA). The immunoreactions were visualized using 3,3’-diaminobenzidine (DAB, Millipore/Merck KGaA, Germany) as a chromogen. Negative controls were prepared identically except for the omission of the primary antibodies.

Two regions of interest were chosen to quantify the activation of microglia and astrocytes: the hippocampus (the object of volumetric study) and the SN (the region of quantification of TH-positive cells). To quantify Iba1 (microglia) or GFAP (astrocyte) immunoreactivity, three microscopic images from the hippocampus and three microscopic images from the SN were randomly captured in different sections at 40x magnification using a light microscope (Zeiss Axio Imager Z1). The images were obtained on the same day with the same intensity of light. The percentage of the area of the whole image containing Iba1 or GFAP immunoreactivity was quantified using ImageJ software (http://imagej.nih.gov/ij/). The results were averaged for each animal and area and used for the statistical analysis.

Stereological counts of TH-positive cell bodies in the substantia nigra pars compacta (SNpc) were obtained using an optical fractionator method. in Stereo Investigator software (MBF Bioscience, Inc., USA) and a light microscope (Olympus BX51). SNpc was outlined at low magnification (2x objective), with reference to a stereological atlas of the rat brain [29]. Nine to ten sections containing SNpc from the 1-in-6 series were analyzed from each brain. Clearly stained cells were counted at higher magnification (60x objective), only if they did not intersect forbidden lines. The counting variables were as follows: sampling grid 200×200μm, counting frame 75×75μm, disector height 8μm, guard zone 2 μm. The coefficient of error in all samples was below 0.1.

### Statistical analysis

All data are presented as means ± S.E.M. Neurotransmitters and metabolites from brain tissue and plasma were analysed using the T-test for independent samples. Using Bonferroni correction the significance was set as a p-value <0.001. The Pearson correlation was used where appropriate. Animal weights, Iba1 and GFAP immunoreactivity, hippocampal volume and the number of TH-positive cells in the SNpc were analysed using the Mann-Whitney U-test. Significance was set as a p-value <0.05. Statistical analyses were performed using Statistica 9.0 software (Statsoft).

## Results

### Brain and plasma levels of monoamines and their metabolites

We found significantly increased levels of DOPAC, HVA and 5-HIAA and decreased levels of 5-HT and 3-MT in each measured brain area in LPS treated animals compared with the control group. DA levels were significantly increased in the striatum and prefrontal cortex and had a trend towards an increase in the hippocampus. The plasma levels of DA and its metabolites followed the brain levels with the exception of 3-MT, which was elevated in the plasma of LPS treated animals. LPS treatment had no effect on plasma levels of 5-HT, but there was a trend for higher plasma levels of its metabolite 5-HIAA. Detailed results are included in Table 1.

**Table 1:** Brain and plasma levels of monoamines and their metabolites.

		**Striatum**	**Hippocampus**	**Prefrontal cortex**	**Plasma**
	Control	51.13±1.10	32.14±1.67	23.81±0.69	160.45±3.70
**DA**	LPS	80.61±2.79	38.52±1.00	33.26±1.33	213.55±4.51
	T-test	t(41) = -10.65, p<0.001	t(41) = -3.07, p<0.01	t(41) = -6.68, p<0.001	t(41) = -9.19, p<0.001
	Control	15.43±0.50	24.91±0.52	16.03±0.46	7.42±0.19
**DOPAC**	LPS	25.40±1.08	29.30±0.77	22.55±0.78	8.95±0.13
	T-test	t(41) = -8.97, p<0.001	t(41) = -4.91, p<0.001	t(41) = -7.52, p<0.001	t(41) = -6.39, p<0.001
	Control	7.45±0.27	16.81±0.53	14.84±0.69	4.94±0.18
**3-MT**	LPS	4.53±0.17	14.02±0.38	9.28±0.60	7.90±0.30
	T-test	t(41) = 8.62, p<0.001	t(41) = 4.07, p<0.001	t(41) = 5.91, p<0.001	t(41) = -8.74, p<0.001
	Control	12.76±0.38	27.79±1.26	18.77±0.66	5.69±0.28
**HVA**	LPS	21.70±0.69	38.35±1.94	25.73±0.70	9.23±0.24
	T-test	t(41) = -11.88, p<0.001	t(41) = -4.73, p<0.001	t(41) = -7.19, p<0.001	t(41) = -9.36, p<0.001
	Control	24.80±0.77	33.47±1.50	25.30±0.60	46.33±2.04
**5-HT**	LPS	18.62±1.12	22.54±0.90	19.53±0.59	41.89±1.79
	T-test	t(41) = 4.68, p<0.001	t(41) = 5.84, p<0.001	t(41) = 6.75, p<0.001	t(41) = 1.59, p = 0.12
	Control	13.59±0.69	17.51±0.48	22.67±1.00	230.50±3.53
**5-HIAA**	LPS	20.00±0.85	22.96±1.57	30.29±0.98	245.32±2.35
	T-test	t(41) = -5.91, p<0.001	t(41) = -3.64, p<0.001	t(41) = -5.36, p<0.001	t(41) = -3.30, p<0.01

Values (ng/ml) represent mean ± SEM.

### Brain and plasma levels of glutamate and GABA

There were significantly increased levels of GLU in each measured brain area, decreased levels of GABA in the hippocampus and a decreasing trend of GABA in the prefrontal cortex in LPS treated animals compared with the control group. The plasma levels of GLU and GABA were both significantly elevated in LPS treated animals. Detailed results are included in Table 2.

**Table 2:** Brain and plasma levels of glutamate and γ-aminobutyric acid.

		**Striatum**	**Hippocampus**	**Prefrontal cortex**	**Plasma**
	Control	34.46±0.81	29.25±0.94	30.30±0.58	13.86±0.35
**GLU**	LPS	46.31±1.23	42.49±1.48	40.85±1.09	18.02±0.41
	T-test	t(41) = -8.32, p<0.001	t(41) = -7.85, p<0.001	t(41) = -9.08, p<0.001	t(41) = -8.07, p<0.001
	Control	5.32±0.39	6.28±0.21	5.98±0.16	9.01±0.14
**GABA**	LPS	6.01±0.42	3.62±0.14	4.97±0.37	12.47±0.30
	T-test	t(41) = -1.20, p = 0.24	t(41) = 9.99, p<0.001	t(41) = 2.73, p<0.01	t(41) = -11.21, p<0.001

Values (ng/ml) represent mean ± SEM.

### Brain and plasma levels of tryptophan and kynurenine pathway metabolites

We detected significantly increased levels of TRP, KYN, 3-OH-KYN and QUIN in each measured brain area and plasma in LPS treated animals compared with the control group. The LPS treatment had no effect on KYNA levels. Detailed results are included in Table 3.

**Table 3:** Brain and plasma levels of tryptophan and kynurenine pathway metabolites.

		**Striatum**	**Hippocampus**	**Prefrontal cortex**	**Plasma**
	Control	6.63±0.26	6.25±0.27	5.95±0.27	4.09±0.11
**TRP**	LPS	20.17±1.08	20.46±1.70	21.57±1.57	6.67±0.24
	T-test	t(41) = -13.48, p<0.001	t(41) = -9.25, p<0.001	t(41) = -10.98, p<0.001	t(41) = -10.34, p<0.001
	Control	63.93±1.00	75.59±1.66	66.99±0.89	65.13±1.40
**KYN**	LPS	111.83±1.89	107.46±2.86	109.86±1.48	94.58±1.83
	T-test	t(41) = -23.76, p<0.001	t(41) = -10.10, p<0.001	t(41) = -25.94, p<0.001	t(41) = -12.99, p<0.001
	Control	23.15±2.13	23.18±2.11	23.23±2.08	1.89±0.08
**KYNA**	LPS	23.89±2.95	23.01±3.15	22.23±3.08	1.80±0.10
	T-test	t(41) = -0.20, p = 0.84	t(41) = 0.05, p = 0.96	t(41) = 0.28, p = 0.78	t(41) = 0.71, p = 0.48
	Control	17.81±0.74	21.18±0.71	19.30±0.60	47.88±0.72
**3-OH-KYN**	LPS	36.20±1.15	40.48±0.75	37.10±1.26	75.21±2.03
	T-test	t(41) = -13.96, p<0.001	t(41) = -18.50, p<0.001	t(41) = -13.59, p<0.001	t(41) = -13.84, p<0.001
	Control	32.84±0.72	32.33±0.52	32.35±0.61	16.65±0.43
**QUIN**	LPS	43.41±0.46	41.05±0.78	40.43±0.96	22.24±0.46
	T-test	t(41) = -11.88, p<0.001	t(41) = -9.38, p<0.001	t(41) = -7.33, p<0.001	t(41) = -8.83, p<0.001

Values (ng/ml) represent mean ± SEM.

### Animal weights

We did not find any significant weight reduction at PD 93–97 in LPS treated animals (401±11g) compared with the control group (413±18 g) [U(14) = 23, Z = 0.89, p = 0.37].

### Activation of microglia and astrocytes

**Figure 2:**
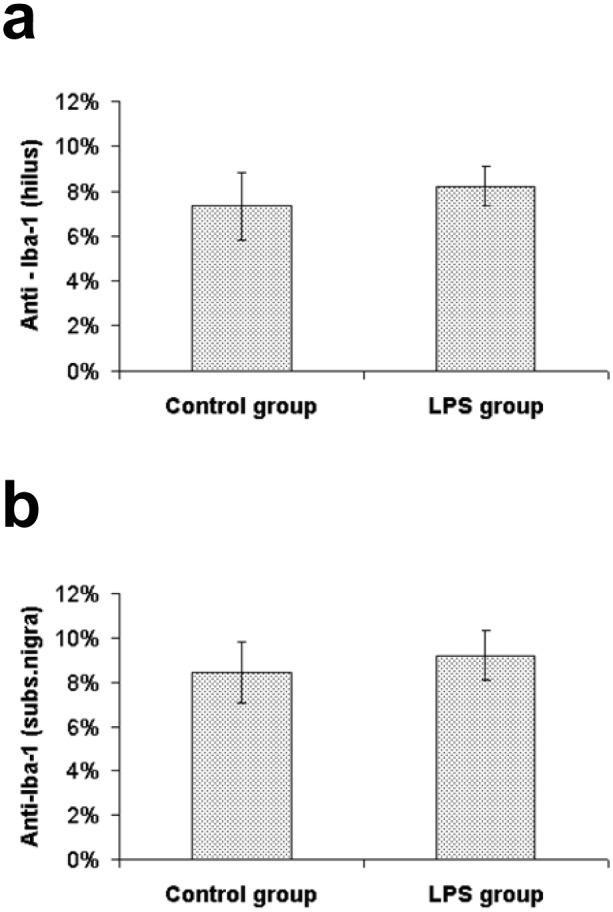
Percentage of the area containing Iba1 immunoreactivity. Values (%) represent mean ± SEM. a) hilus of the dentate gyrus, b) substantia nigra.

Microglia marked by the Iba1 antibody were observed in the whole brain in both LPS treated animals and saline treated controls. In both tested groups we found Iba1-positive cells with a small round to oval cell body and long thin ramified branches. Hyperthrophied microglia with an atypical shape of the cell body and/or thicker processes which represent a transitional stage to the activated microglia were occasionally found in both groups. The percentage of the area containing Iba1 immunoreactivity in the hippocampus of LPS treated animals (8.25±0.89%) did not differ from the control group (7.35±1.5%) [U(14) = 26, Z = -0.58, p = 0.56]. We also did not reveal any difference in the SNpc between LPS treated animals (9.23±1.1%) and the control group (8.46±1.37%) [U(14) = 26, Z = -0.58, p = 0.56] (Fig. 2).

**Figure 3:**
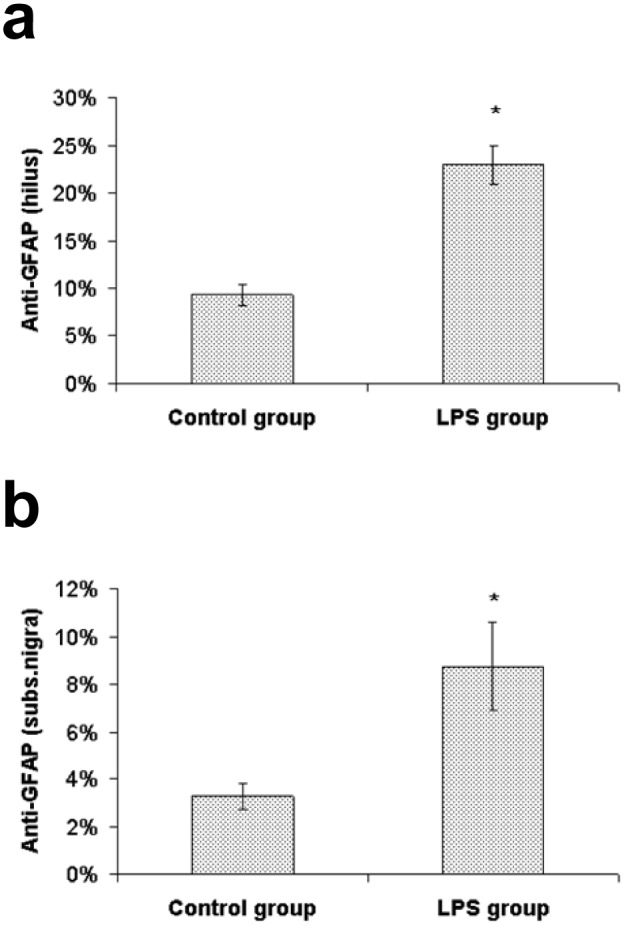
Percentage of the area containing GFAP immunoreactivity. Values (%) represent mean ± SEM (* indicates p<0.05 from the control group). a) hilus of the dentate gyrus, b) substantia nigra.

**Figure 4:**
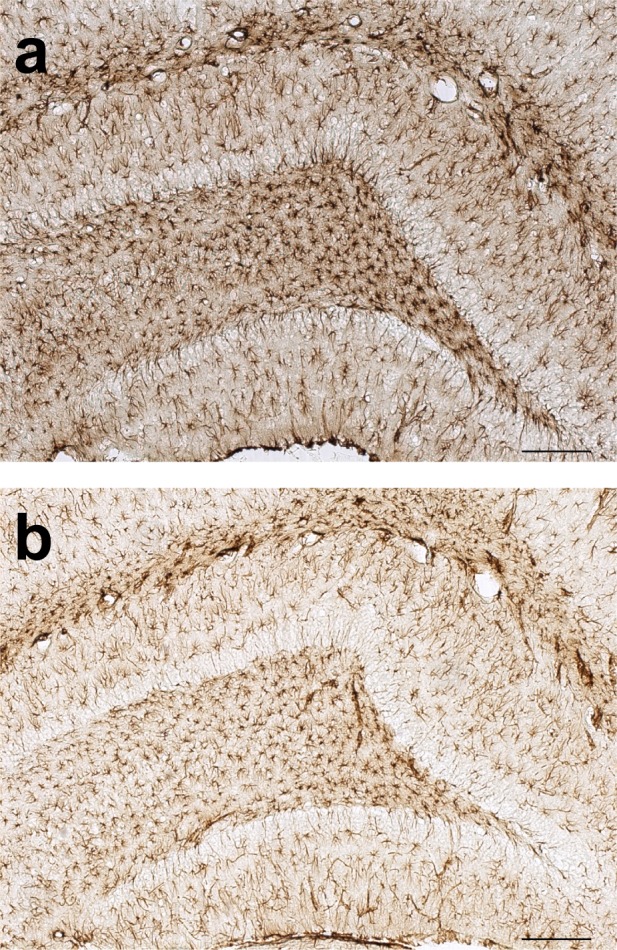
Microphotographs of GFAP immunoreactivity in the dentate gyrus (scale bar = 200μm). a) LPS treated group, b) Control group.

Astrocytes marked by the GFAP antibody were localized in the whole brain in both LPS treated animals and saline treated controls. However, the morphological characteristic of GFAP-positive cells was different in LPS treated animals (hypertrophied cell body and thicker processes) compared to those in the control group. Although the astroglial hypertrophy was present in multiple brain regions (especially in periventricular areas, but less so in the cortex), the most distinct changes were found in the hippocampus (hilus of the dentate gyrus, stratum lacunosum moleculare and stratum oriens) and in the SNpc. The percentage of the area containing GFAP immunoreactivity of LPS treated animals was significantly higher than in the control group in the hippocampus (22.97±2.02% vs. 9.26±1.11%) [U(6) = 0, Z = -2.17, p<0.05] and in the SNpc (8.75±1.85% vs. 3.25±0.56%) [U(6) = 0, Z = -2.17, p<0.05] (Figs. 3 and 4).

### Neurodegeneration

We did not detect any Fluoro-Jade B positivity at PD 93–97 in the brains of animals perinatally treated with LPS. The analysis of cell nuclei did not show any sign of nuclear pathology, e.g. nuclear shrinkage or fragmentation. The saline treated controls exhibited the same results.

### Hippocampal volume

**Figure 5:**
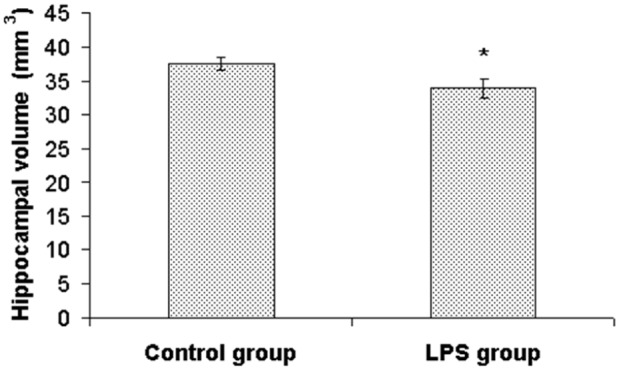
Effect of perinatal LPS treatment on hippocampal volume. Values (mm^3^) represent mean ± SEM (* indicates p<0.05 from the control group).

We found a significant reduction of hippocampal volume in LPS treated animals (33.9±1.4 mm^3^) compared with the control group (37.6±0.9 mm^3^) [U(14) = 11.5, Z = 2.1, p<0.05]. The reduction of hippocampal volume in LPS treated animals was approximately 10% (Fig. 5).

### Tyrosine hydroxylase-positive cells in the substantia nigra, pars compacta

**Figure 6:**
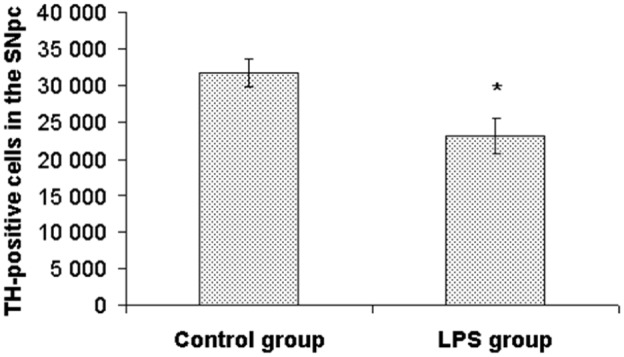
Number of TH-positive cells in the substantia nigra, pars compacta. Values represent mean ± SEM (* indicates p<0.05 from the control group).

There was a significant reduction of the number of TH-positive cells in the SNpc in LPS treated animals (23 172±2 438) compared with the control group (31 830±1 891) [U(13) = 9, Z = 2.14, p<0.05]. The reduction of TH-positive cells in LPS treated animals was approximately 27% (Fig. 6).

## Discussion

The main findings of this study are changes in the biochemistry and brain morphology of adult animals perinatally exposed to LPS. Our study showed that the early postnatal LPS administration led to a volume reduction of the hippocampus. To our knowledge this is the first study which documents that the hippocampal volume can be significantly altered not only after intracerebral injection [30] but also after systemic LPS administration, which is a condition more likely to simulate the perinatal infection. The early postnatal period in rats corresponds approximately with the third trimester of human brain development [31, 32]. Over-expression of cytokines during this period disrupts the development of nervous system [33]. Structural brain changes in the adult brains (white matter damage, dilatation of lateral ventricles, striatal volume reduction) resulting from early postnatal LPS exposure have been attributed to the maldevelopment of oligodendrocytes [2, 5, 20, 30, 34], which may in turn underlie maldevelopment of brain networks and even whole brain structures, e.g. hippocampus. Hippocampal volume reduction is one of the signs associated in humans with various neuropsychiatric disorders, such as schizophrenia, major depressive disorder, bipolar disorder, posttraumatic stress disorder, Alzheimer’s disease, Parkinson’s disease or vascular dementia [23–27].

The increased levels of KYN and metabolites from the inflammatory part of the kynurenine pathway (3-OH-KYN, QUIN) in the brain and plasma could be mediated by indole-2,3-dioxygenase (IDO) and the enzymes of the inflammatory part of the kynurenine pathway, which are activated by pro-inflammatory cytokines [22]. The persistent elevation of pro-inflammatory cytokines in adulthood has been described previously by our group in this animal model of early immune stimulation [12]. Unlike KYN and 3-OH-KYN, QUIN is not able to cross the blood brain barrier (BBB) [35]; therefore the production of QUIN in the brain is regulated by local enzyme activity. Hippocampal formation is very sensitive to QUIN induced excitotoxicity [36], which is consistent with our finding of decreased hippocampal volume in LPS treated animals. However, we did not detect any Fluoro-Jade B positivity or changes of neuronal nuclei typical for cell death at PD 93–97, which indicates a lack of ongoing neurodegeneration. This corresponds with the results of studies that did not detect neurodegeneration in adult LPS treated animals hours or weeks after LPS administration [37, 38]. The activation of the inflammatory part of the kynurenine pathway has been described in relation with major depressive disorder, Alzheimer’s disease, Parkinson’s disease, Huntington’s disease and epilepsy [22]. On the other hand, elevated KYN levels have been found in the post-mortem prefrontal cortex [39] and elevated KYN and KYNA levels in cerebral spinal fluid [40] of schizophrenic patients. Although we identified increased levels of KYN in LPS treated animals, we did not find any change of KYNA levels in the brain or plasma compared to the controls.

The higher TRP plasma levels in the LPS group represent an interesting and unexpected finding. Neuropsychiatric disorders such as depression, anxiety and sometimes schizophrenia are usually associated with low TRP levels, which result in a decrease of 5-HT synthesis [41–44]. On the other hand, several studies described increased TRP levels in blood, cerebrospinal fluid and post-mortem brain tissue of schizophrenic patients [45–47]. TRP, an essential amino acid, is absorbed from the diet in the small intestine by a specialized transporter protein and transported in the blood bound to albumin or in a free form, which passes though the BBB [48, 49]. We suggest that the discrepancies in TRP levels in LPS animals might be due to gastrointestinal disturbances, which would change the absorption of TRP from the diet, or by an alteration in albumin-TRP binding, which would increase the plasma levels of free TRP, or by an impairment of TRP catabolism. However the latter is less likely because the immune response activates enzymes of inflammatory part of kynurenine pathway, which results in a decline of TRP and subsequently also 5-HT levels [50]. IDO, the crucial enzyme of kynurenine pathway, catalyzes not only TRP degeneration but also the production of formyl 5-HT from 5-HT [51, 52], which might explain the low levels of 5-HT in the LPS treated animals despite the increased levels of TRP. Alternative explanation of decreased 5-HT levels could be based on enhanced metabolization of 5-HT to 5-HIAA by monoamino oxidase (MAO). In a recent study, the quantification of hippocampal neurotransmitters in a mice model of depression showed an elevated ratio of 5-HIAA to 5-HT reflecting enhanced turnover of 5-HT and increased serotonergic activity in depressive mice [53]. Congruently, in our study, there was a negative correlation between the levels of 5-HT and 5-HIAA (r = -0.69, p = 0.001) in the hippocampus of the LPS treated animals but not in the control animals.

Perinatal LPS exposure has been shown to suppress TH immunoreactivity in the SN and VTA in adulthood [19, 20, 54], which is consistent with our finding of a reduced number of TH-positive cells in the SNpc. However, we also found increased DA levels in the brain and plasma in LPS treated animals. LPS challenge during the perinatal period induces an increase in TH phosphorylation and activity in adrenals persisting until adulthood [16, 55]. TH catalyzes the conversion of amino acid L-tyrosine into DA precursor L-3,4-dihydroxyphenylalanine (L-DOPA), which is able to cross the BBB. We propose that the elevated brain levels of DA in LPS treated animals were evoked by increased L-DOPA synthesis in the periphery and its subsequent crossing through the BBB. Schizophrenia is associated with hyperactivity of mesolimbic dopaminergic neurons and prefrontal hypodopaminergia [56]. High DA levels in the striatum and hippocampus may support early immune stimulation being an animal model of schizophrenia, however the decreased TH immunoreactivity in the SNpc could be more relevant to the pathogenesis of Parkinson’s disease [19, 20, 54].

The increase of plasma levels of DA metabolites (DOPAC, 3-MT, HVA) in LPS treated animals may be associated with increased DA availability in plasma. The increased brain levels of DOPAC and HVA may be explained by the activation of mooaminooxidase B demonstrated in proliferating and reactive astrocytes [57], which were also found in this study.

The reduction of TH expression in the SN and the ventral tegmental area in adult animals after postnatal LPS exposure has been ascribed to a chronic microglial activation in this region [19, 20, 54]. In our study we chose Iba1 as a microglial marker that has proven most helpful in visualizing microglia with details of their processes and which expression increases with microglial activation [58]. However, the microglia in the SN and hippocampus mostly showed signs of a resting state with an occasional occurrence of a hyperthrofied microglial body in both the LPS and control groups. According to some studies intracerebral as well as systemic postnatal LPS administration leads to microglial activation, which persists until adulthood [15, 30, 34, 59]. In contrast, a recent study showed that early postnatal systemic LPS administration (PD5) increased microglial density in the hippocampus 48 hours after the administration, but in PD21 and PD74 animals the microglial density and the number of microglia did not differ from saline treated controls [21]. The authors also speculated on the possible existence of a target or maximum density of microglia in the brain [21].

Astrocytes influence a series of postnatal developmental steps, e.g. neuronal synapse formation, myelination and homeostasis of the central nervous system [60–62]. The transition of astrocytes into a reactive state, as we found in the SN and hippocampus of LPS treated animals, can possibly interfere with its functions because reactive astrocytes in vitro produce neurotoxic molecules such as NO, reactive oxygen species and TNFα [63]. On the other hand, pro-inflammatory mediators produced by microglia support neuroprotective astrocyte responses [64, 65]. Astrogliosis was reported in patients with schizophrenia, autism and neurodegenerative disorders such as Alzheimer’s or Parkinson’s disease [66–69]. In contrast, major depressive disorder is characteristic of the reduction in the density of astrocytes [70].

Proinflammatory mediators induce the synthesis of QUIN, which acts as an NMDA receptor agonists and an important neurotoxic compound [22]. At high levels, QUIN promotes GLU release by neurons, inhibits its reuptake by astrocytes and inhibits astroglial glutamine synthetase (GS) resulting in an accumulation of GLU [35], as was found in the present study. GLU serves as a precursor for the major inhibitory neurotransmitter GABA. Our finding of decreased GABA levels in the hippocampus and prefrontal cortex in the LPS group may be mediated by the inhibition of GS [35]. The inhibition of GS is also induced by reactive astrogliosis [71]. The increased plasma levels of GABA in the LPS group may be due to synthesis from GLU in peripheral tissues [72, 73]. In our study there was a negative correlation between the levels of GLU and GABA (r = -0.51, p = 0.03) in the hippocampus of LPS treated animals but not in control animals. Abnormalities in glutamatergic and GABAergic systems such as the combination of increased GLU and decreased GABA levels in the brain have been associated with mood and anxiety disorders or autism [74–77].

In conclusion, the early immune stimulation induced by postnatal systemic administration of LPS leads to biochemical actions resulting in alterations of neurotransmitter metabolism and activation of the kynurenine pathway of tryptophan metabolism as well as astrogliosis, hippocampal volume reduction and a decrease of tyrosine hydroxylase immunoreactivity in the substantia nigra. Our results suggest a pathogenetic link between early immune stimulation and neuropsychiatric disorders such and schizophrenia, mood disorders, anxiety disorders, autism, Parkinson’s disease and Alzheimer’s disease.

## Supporting Information

S1 ARRIVE Guidelines Checklist (DOC)
